# Changes in Bacterial Endophyte Community Following *Aspergillus* *flavus* Infection in Resistant and Susceptible Maize Kernels

**DOI:** 10.3390/ijms22073747

**Published:** 2021-04-03

**Authors:** Rajtilak Majumdar, Shyam L. Kandel, Jeffrey W. Cary, Kanniah Rajasekaran

**Affiliations:** Food and Feed Safety Research Unit, Southern Regional Research Center, United States Department of Agriculture, Agricultural Research Service, New Orleans, LA 70124, USA; raj.majumdar@usda.gov (R.M.); shyam.kandel@usda.gov (S.L.K.)

**Keywords:** *Aspergillus flavus*, microbiome, metagenomics, disease resistance, endophyte, aflatoxins, mycotoxins, maize, plant-pathogen interaction

## Abstract

*Aspergillus flavus* (*A. flavus*)-mediated aflatoxin contamination in maize is a major global economic and health concern. As *A. flavus* is an opportunistic seed pathogen, the identification of factors contributing to kernel resistance will be of great importance in the development of novel mitigation strategies. Using V3–V4 bacterial rRNA sequencing and seeds of *A. flavus*-resistant maize breeding lines TZAR102 and MI82 and a susceptible line, SC212, we investigated kernel-specific changes in bacterial endophytes during infection. A total of 81 bacterial genera belonging to 10 phyla were detected. Bacteria belonging to the phylum *Tenericutes* comprised 86–99% of the detected phyla, followed by *Proteobacteria* (14%) and others (<5%) that changed with treatments and/or genotypes. Higher basal levels (without infection) of *Streptomyces* and *Microbacterium* in TZAR102 and increases in the abundance of *Stenotrophomonas* and *Sphingomonas* in MI82 following infection may suggest their role in resistance. Functional profiling of bacteria using 16S rRNA sequencing data revealed the presence of bacteria associated with the production of putative type II polyketides and sesquiterpenoids in the resistant vs. susceptible lines. Future characterization of endophytes predicted to possess antifungal/ anti-aflatoxigenic properties will aid in their development as effective biocontrol agents or microbiome markers for maize aflatoxin resistance.

## 1. Introduction

Mycotoxin contamination in food and feed crops poses a serious threat to humankind worldwide. Four fungal genera, *Aspergillus*, *Fusarium*, *Penicillium*, and *Alternaria*, are major producers of mycotoxins in crop plants. Among them, *Aspergillus flavus* has the greatest negative impact on crop losses and human/livestock health [1,2,3]. Oilseed crops such as maize and peanut are crops commonly infected by *A. flavus* and subsequently contaminated with carcinogenic aflatoxins and numerous other toxic secondary metabolites (SMs). Other crops such as rice, tree nuts, figs, spices, and dried fruits are also prone to aflatoxin contamination either pre- or post-harvest (reviewed in [4,5]). Computer models of future climate change scenarios predict significant increases in aflatoxin contamination due to environmental conditions more favorable for *A. flavus* growth and survival [6].

Maize is a major staple food and feed crop in the world. In 2018–2019, the global acreage of maize was estimated to be about 197.2 million hectares, producing approximately 1.09 billion metric tons. While economic losses due to aflatoxin contamination of maize varies from year to year and are hard to quantify, computer models of alterations in future climatic conditions in the U.S. predict aflatoxin contamination could result in annual losses to the corn industry ranging from $52.1 million to $1.6 billion USD [2]. Consumption of aflatoxin-contaminated foods and feeds can result in severe health impacts in humans and livestock. Ingestion of food contaminated with aflatoxins over a long period can result in chronic adverse health impacts such as liver cancer in adults and stunted growth in children. In addition, *A. flavus* infection causes aspergillosis in individuals who are immunocompromised (reviewed in [7]). Several aflatoxin mitigation strategies have been developed. The most effective pre-harvest strategy implemented to date on a global scale is the use of non-aflatoxigenic *A. flavus* biological control strains [7,8]. Despite years of demonstrated significant reduction in aflatoxin levels in treated field crops, the application of non-aflatoxigenic biocontrol formulations often does not result in reductions in aflatoxin contamination below that set by regulatory agencies for allowable levels in foods and feed commodities. For this reason, additional pre-harvest control strategies are being developed to complement biocontrol including novel application strategies and formulations of non-aflatoxigenic *A. flavus* biocontrol isolates [9], improved agronomic practices, conventional and marker-assisted breeding, transgenic expression of natural or synthetic antifungal genes, and RNAi-mediated host-induced gene silencing (HIGS) of critical fungal genes required for pathogenesis and aflatoxin production [7,10]. As *A. flavus* is a kernel-specific pathogen in maize, the identification of host kernel-specific endophytes with potential anti-aflatoxigenic/antifungal properties will be of great resource towards the development of additional biocontrol agents that do not produce other potentially toxic secondary metabolites and potential interactions with indigenous soil microbes [11].

The host plant microbiome, in this regard, can play an important role in overall resistance to pathogens. Several studies have demonstrated the role of host plant microbiome in resistance against a wide variety of pathogens including fungi, bacteria, viruses, etc. in addition to their positive influence against abiotic stress [12]. Beneficial microbes contribute to host plant resistance by producing secondary metabolites that have direct antimicrobial properties against target pathogens/pests, or metabolites produced by endophytes can modulate host plant defense-related pathways, thereby contributing to resistance (reviewed in [13,14]). Seed microbiomes likely to serve as the starting inoculum for the plant microbiomes are crucial for plant health and resilience [15]. However, limited information is available on the potential role of maize bacterial endophytes against mycotoxigenic fungi. The bacterial endophyte *Paenibacillus polymyxa*, isolated from wild teosinte maize, was highly potent in suppressing *Fusarium graminearum* growth in vitro and production of the associated mycotoxin, deoxynivalenol (DON [16]). In another study, *Bacillus velezensis* strains isolated from maize kernels demonstrated antagonistic activity against pathogenic strains (measured by radial growth) of *Talaromyces funiculosus*, *Penicillium oxalicum*, and *Fusarium verticillioides* [17]. Though some information is available on the role of maize kernel-specific endophytes against *Fusarium*, no information is available on the potential role of bacterial endophytes against *A. flavus* during seed infection.

An ongoing study by our group aimed at elucidating the mechanisms regulating gene expression in maize kernels in response to *A. flavus* infection (using the same resistant TZAR102 and MI82 maize lines used in the present study) showed the presence of a small percentage of transcripts of bacterial origin. A closer inspection of the data revealed that bacterial genera initially thought to be contaminants in the resistant but not susceptible maize genotypes represent known endophytes with demonstrated antimicrobial/antipathogenic properties. We hypothesized that specific endophytes in the kernels of the resistant maize genotypes may contribute to overall kernel resistance against *A. flavus* and aflatoxin accumulation during kernel infection. Aflatoxin-resistant TZAR102 was developed by United States Department of Agriculture—Agricultural Research Service (USDA-ARS) in collaboration with the International Institute of Tropical Agriculture, Nigeria [18], using a moderately resistant U.S. line MI82 [19] and an African line 1386 as parents. As little is known with respect to the temporal (early and late infection stages) changes in the maize kernel bacterial endophyte community structure and abundance in response to *A. flavus* infection, we used a bacterial 16S rRNA gene sequencing approach of DNA isolated from kernels to identify any significant differences in microbial community structure in the resistant (TZAR102 and MI82) vs. susceptible (SC212) lines following *A. flavus* infection. Our results indicate distinct shifts in community structure and abundance of the kernel bacterial endophytes upon *A. flavus* infection between aflatoxin-resistant and -susceptible maize genotypes.

## 2. Results

After stringent quality sequence curation, a total of 9,000,000 sequences were parsed and 8,857,967 sequences were mapped to zero-radius operational taxonomic units (zOTUs). A total of 5,164,472 sequences were identified within the Bacteria and Archaea domains and were utilized for final microbiota analyses. The average reads per sample was 114,766. For alpha and beta diversity analysis, the samples were rarefied to 100,000 sequences. The data were evaluated in a multivariate manner to determine the changes between different treatment groups.

### 2.1. Significant Differences Observed in Bacterial Phyla between Resistant and Susceptible Lines

**Figure 1:**
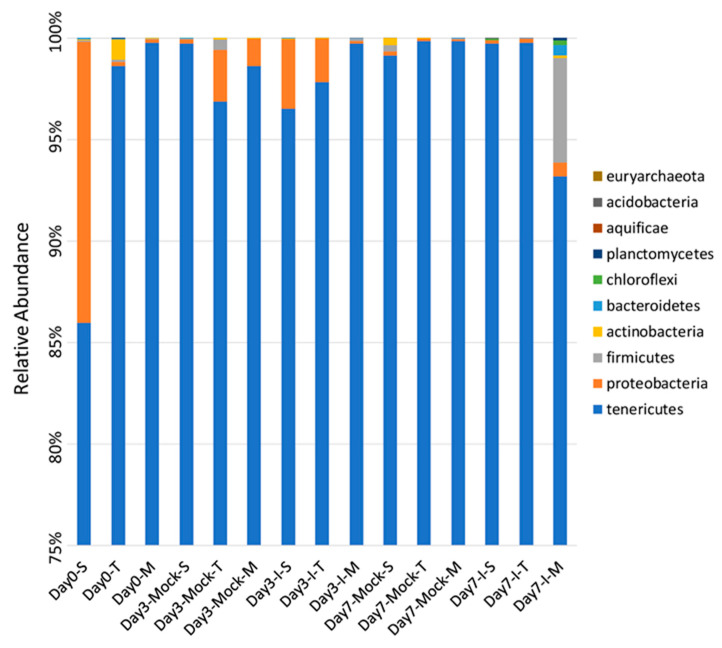
The mean relative abundance of prokaryote phyla in the kernels of *A. flavus*-susceptible and -resistant maize genotypes. S: SC212, susceptible genotype; T: TZAR102, resistant genotype; M: MI82, resistant genotype. Mock = mock control and I = *A*. *flavus*-infected. The data are mean ± standard error (SE) of 3 replicates, and each replicate consists of four seeds.

Overall, a total of 10 different phyla were detected among all samples. At day 0, in both resistant lines TZAR102 and MI82, bacteria belonging to the phylum *Tenericutes* were the predominant ones (≥99%) in comparison to the susceptible line SC212 (86%). The percentage of *Proteobacteria* was much higher (14%) in SC212 at this time point compared to the resistant lines (0.2%; Figure 1). Detailed percentages of bacterial phyla in different treatment groups are presented in Table S1. At day 3, both in the mock and infected kernels of SC212, *Tenericutes* comprised 96–99% of the bacterial community. There was a significant increase (from 0.2% to 3%) in *Proteobacteria* in SC212 in response to *A. flavus* infection (vs. mock control). In the mock kernels of TZAR102 and MI82 at day 3, *Tenericutes* contributed to 96–98% of the bacterial community and a higher (vs. SC212) percentage of *Proteobacteria* (1.3–2.5%) was observed, which decreased significantly in response to fungal infection at this time point. The other bacterial phyla detected in this study were typically below 0.1% abundance in all maize genotypes except for the phyla *Firmicutes*, *Bacteroidetes*, and *Chloroflexi*, which increased to 5.1%, 0.55%, and 0.21%, respectively, only in the infected kernels of MI82 at day 7 (Figure 1).

### 2.2. Relatively Fewer Bacterial Genera Varied between Treatment Groups

Based on the relative abundance of target-specific genera identified, statistical comparisons were performed using ANOVA and post hoc pairwise comparisons were made using Tukey’s test. We evaluated whether any specific genera were significantly different between treatment groups. A total of 81 different bacterial genera were detected among all samples. There were relatively fewer genera including *Stenotrophomonas*, *Sphingomonas*, *Streptomyces*, *Microbacterium*, *Buchnera*, and *Candidatus* that were significantly different between treatment groups (resistant vs. susceptible/mock vs. infected) (Table S2). At day 0, the relative abundances of both *Streptomyces* and *Microbacterium* in TZAR102 and of *Candidatus* in both TZAR102 and MI82 were significantly higher than that observed in the susceptible line SC212. At day 3, infected kernels of MI82 showed significant increases in *Stenotrophomonas* and *Sphingomonas* abundance compared to the mock control samples of the same line. While infected kernels of SC212 had a higher content of *Buchnera* than the two resistant lines at day 3, *Stenotrophomonas* increased significantly in the infected kernels of the resistant line MI82 compared to the susceptible line at day 7.

### 2.3. Hierarchal Clustering Shows Predominant Genera among Different Treatment Groups

**Figure 2:**
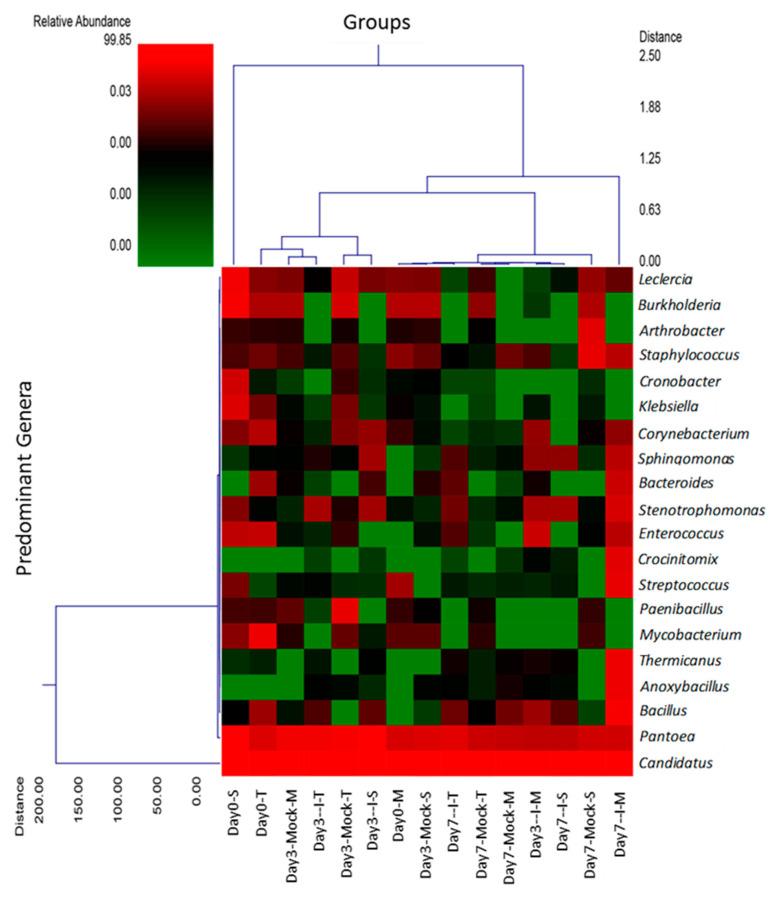
Dual hierarchal dendrogram evaluation of the taxonomic classification data, with each sample clustered on the X-axis labeled based on the treatment. Samples with more similar microbial populations are mathematically clustered closer together. The genera (consortium) are used for clustering. Thus, the samples with a more similar consortium of genera cluster closer together, with the length of connecting lines (top of heatmap) related to the similarity and shorter lines between two samples indicating closely matched microbial consortiums. The heatmap displays the mean relative abundance (percentages) of the top 20 predominant genera for *A. flavus*-susceptible (S: SC212) and -resistant (T: TZAR102; M: MI82) maize genotypes. Mock = mock control and I = *A*. *flavus*-infected. The predominant genera are represented along the right Y-axis. The legend for the heatmap is provided in the upper left corner. The data are mean ± SE of 3 replicates, and each replicate consists of four seeds.

To provide a visual overview combined with analysis, we utilized a dual hierarchal dendrogram to display the data for the predominant genera (Figure 2), with clustering related to the different groups. A higher relative abundance of bacterial genera was represented by *Candidatus*, *Pantoea*, *Stenotrophomonas*, *Bacillus*, and *Leclercia* considering all treatment groups.

### 2.4. Bacterial Diversity Varied with Fungal Infection in the Resistant and Susceptible Lines

#### 2.4.1. Alpha Diversity of Samples

**Figure 3:**
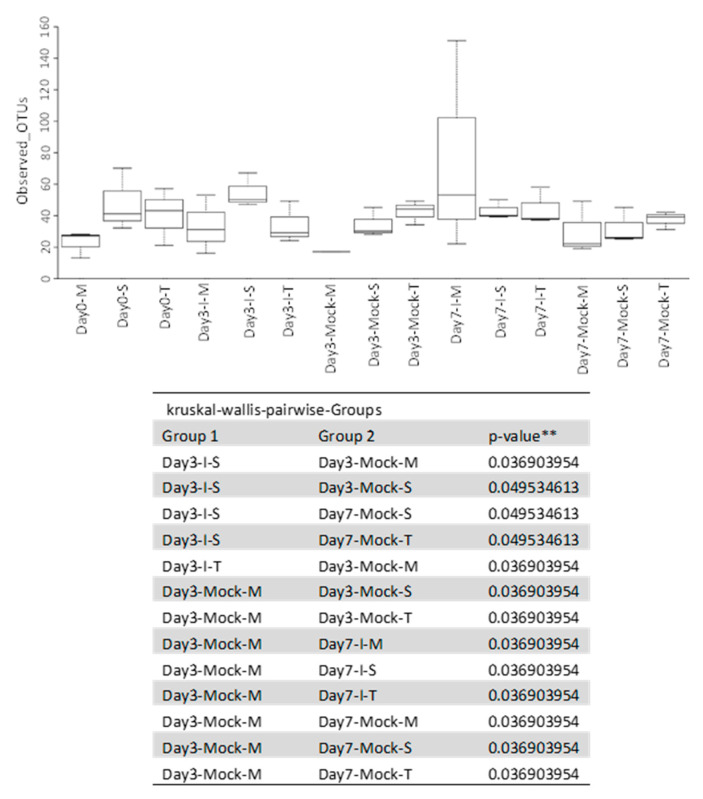
Alpha diversity of samples. Observed OTU boxplot and Kruskal–Wallis pairwise comparisons (** *p <* 0.05). The data are mean ± SE of 3 replicates, and each replicate consists of four seeds.

Alpha diversity is often used to evaluate how many different microbial species are within a given sample or treatment group. Statistical comparisons of the observed operational taxonomic units (OTUs) and Shannon Diversity Indices for each sample group were conducted using Kruskal–Wallis pairwise comparisons (Figure 3). Based on the number of observed OTUs within each line (SC212 (S), TZAR102 (T), and MI82 (M))], the microbial diversity of the inoculated group on day 3 from the susceptible line (day 3-I-S) was significantly greater (*p* < 0.05) than that found in the mock group from the susceptible line collected on the same day (day 3-Mock-S; Figure 3).

Additionally, when comparing the number of observed OTUs in the mock and inoculated groups between susceptible and resistant lines, the microbial diversity of the inoculated group on day 3 from the susceptible line (day 3-I-S) is significantly greater (*p* < 0.05) than that found in the mock group from resistant line M (day 3-Mock-M) collected on the same day (Figure S1).

#### 2.4.2. Beta Diversity of Samples

**Figure 4:**
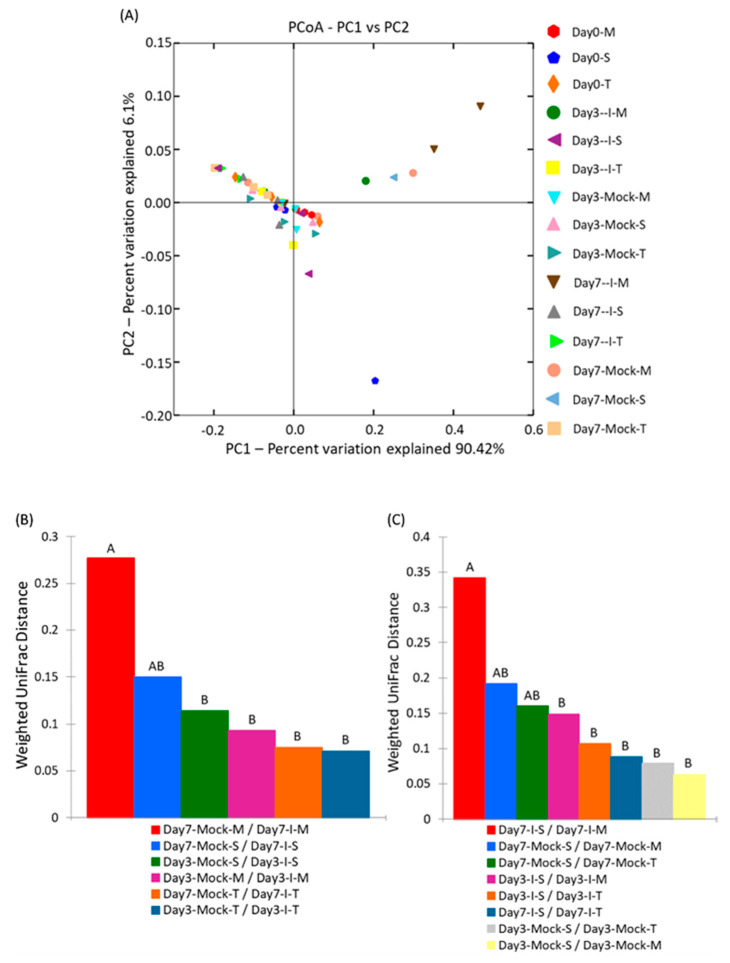
The profile of endophytic bacteria in different samples: (**A**) principal coordinate analysis (PCA), (**B**) comparison of weighted UniFrac distances within each genotype at day 3 and day 7, and (**C**) comparison of weighted UniFrac distances between maize *A. flavus*-susceptible (S) and -resistant genotypes (T and M) at day 3 and day 7. S: SC212, susceptible genotype; T: TZAR102, resistant genotype; M: MI82, resistant genotype. Mock = mock control and I = *A*. *flavus*-infected. Uppercase “A” and “B” above the bars have been used to compare significant difference (*p* < 0.05) among treatments. The data are mean ± SE of 3 replicates, and each replicate consists of four seeds.

Beta diversity allows for a comparison of the communities of microbes, taking into consideration both how many different microbes are in the sample and how these microbes are phylogenetically related. For a comparison of the overall microbial communities among different samples, a beta-diversity analysis was performed using Principal Coordinate Analysis (PCA; Figure 4A) and microbial community structure was analyzed using weighted UniFrac distance matrices (Figure 4B,C). Principal coordinate analysis plots helped to visualize the data in these matrices, and pairwise analysis of similarities (ANOSIM) was utilized to determine if there were any significant differences between the microbial communities. The highest PCA variations observed were 90.4% (PC1) and 6.1% (PC2), suggesting a wide separation between different samples. The infected samples of MI82-resistant line (day 7-I-M) were relatively clustered together in comparison to the rest. A comparison of the weighted UniFrac distances within each genotype at day 3 and day 7 showed significant differences only between day 7 mock and infected samples of the susceptible line SC212 (Figure 4B), whereas a comparison of the weighted UniFrac distances between susceptible (S) and resistant genotypes (T and M) at day 3 and day 7 showed a significant difference between resistant lines (TZAR102 and MI82) and the susceptible line (SC212) only in the mock samples at day 7 (Figure 4C). A comparison of the within-group weighted UniFrac distances did not show any difference (Figure S2).

Linear discriminant analysis effect size (LEfSe) was used to identify potential biomarkers linked to aflatoxin resistance. Qiime2 taxonomic analysis was completed using the greengenes 13_8 database. The relative abundance of taxonomic features was used as input for the LEfSe analysis and was completed using the huttenhower galaxy server. Only 3 treatment groups (day 0-S, day 3-I-M, and day 7-Mock-S) were found to be significantly different (LDA score > 2.0; *p* < 0.05) following the pairwise Wilcoxon test (Figure S3). The bacterial phyla associated with the resistant line, MI82 samples (day 3-I-M), were represented by *Rickettsiales*, *Proteobacteria*, *Mitochondria*, *Alphaproteobacteria*, and *Enterobacteriales*.

### 2.5. Predicted KEGG Pathways Were Highly Abundant in the Resistant Lines

**Figure 5:**
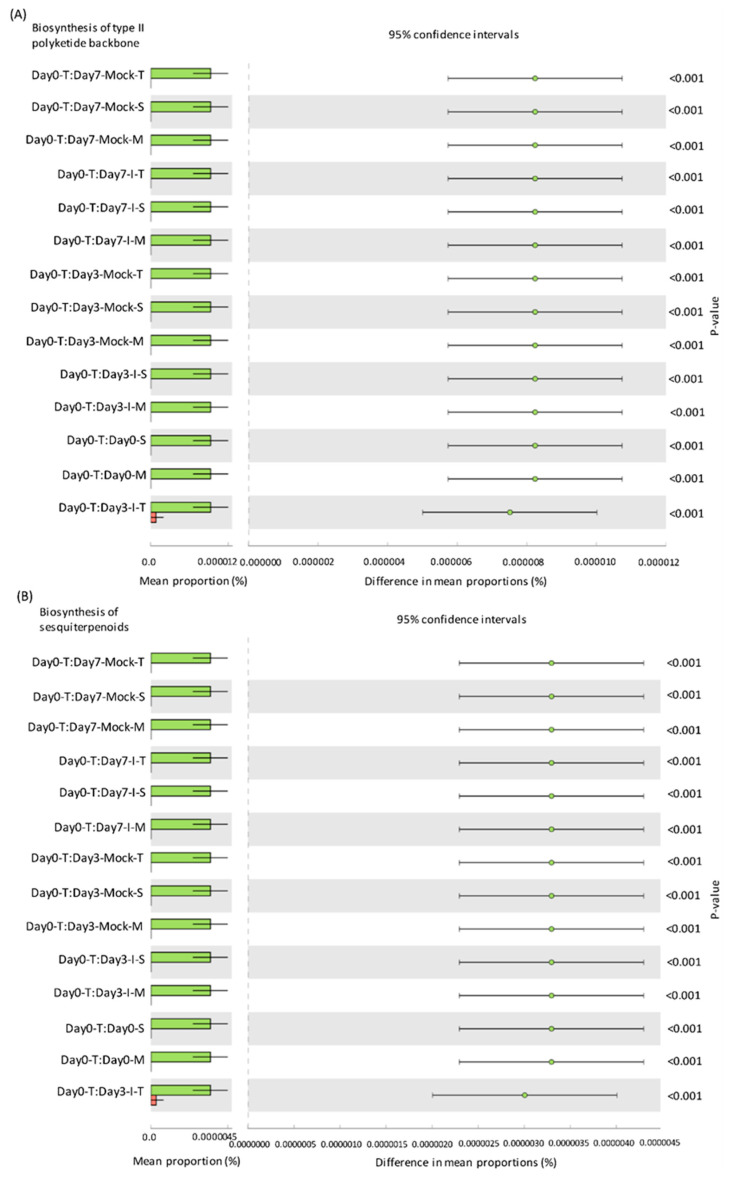
Kyoto Encyclopedia of Genes and Genomes (KEGG) modules found to be significantly different (*p* < 0.05) between treatment groups after Bonferroni test correction: (**A**) biosynthesis of the type II polyketide backbone and (**B**) sesquiterpenoid biosynthesis. Note: The expression of each KEGG module is significantly reduced between Day0-T (Green) and all other treatment groups. S: SC212, susceptible genotype; T: TZAR102, resistant genotype; M: MI82, resistant genotype. Mock = mock control and I = *A*. *flavus*-infected. The data are mean ± SE of 3 replicates, and each replicate consists of four seeds.

To determine if any correlations exist between bacterial communities associated with resistant maize genotypes and the possible functional biological roles with respect to resistance mechanisms that they may impart to the plant host, functional profiling of microbial communities was performed. The Kyoto Encyclopedia of Genes and Genomes (KEGG) pathways that were significantly different among treatment groups were putatively involved in the biosynthesis of type II polyketides, sesquiterpenoids, and neuroactive ligand–receptors in the resistant and susceptible lines (Figure 5 and Figure S4). Both resistant lines showed significantly higher representation of bacteria harboring these pathways in comparison to the susceptible line at the basal level, i.e., in the absence of any fungal infection.

## 3. Discussion

Beneficial microbes (bacteria, fungi, viruses, etc.) in host plants contribute to host resistance against various pathogens, pests, and abiotic stress (reviewed in [12,20]). Their relative abundance varies with crop species, tissue type, stressors, and developmental stages. As endophytes reside inside the plant cells and can colonize at the appropriate cell/tissue types, they may provide additional advantages over traditional biocontrol agents with respect to efficacy in controlling pathogens and pests at the infection sites. Maize is an important food and feed crop grown worldwide. Rampant use of synthetic chemicals for disease control in plants and gradual development of resistance against these chemicals are greatly alarming. Future use of endophytes as potential biocontrol agents against target pathogen(s) requires the identification of specific endophyte candidates associated with a disease resistance trait followed by their functional characterization. Maize is susceptible to *A. flavus* infection and aflatoxin contamination, leading to significant adverse health and economic impacts. An earlier study by our group using the same *A. flavus*-resistant maize lines (as in the current study) detected the presence of bacterial transcripts from known endophytes in the infected kernels. The current study was undertaken to understand how the kernel microbiome responds to *A. flavus* infection in a temporal manner and if there are any potential microbial markers that may be associated with aflatoxin resistance in the resistant lines.

The predominant maize kernel bacteria identified in our current study belong to the phyla *Tenericutes* that comprised 96–99% of the bacterial population among all genotypes. This was followed by *Proteobacteria*, which varied widely between 1.3–14% among different treatment groups, and the remaining phyla represented below 0.1% (Figure 1 and Table S2). The relative abundance of the bacterial phyla in maize kernels in this study are in line with earlier observations [21], but our work shows significant increases in bacterial endophytes belonging to the phyla *Firmicutes*, *Bacteroidetes*, and *Chloroflexi* in the resistant line (MI82) in response to *A. flavus* infection. This may indicate a potential role of these phyla in the defense response against *A. flavus*. Our previous study showed that fungal load and aflatoxin production were significantly lower in the resistant lines at early (day 3) and late (day 7) infection stages [22]. Specifically, the MI82 line showed less fungal colonization on the seed surface and accumulated the lowest amount of aflatoxins both at the early and late infection stages [22]. At the genus level, the bacterial genera that showed significant differences in abundance among different treatment groups (resistant vs. susceptible/mock vs. inoculated) belonged to *Stenotrophomonas*, *Sphingomonas*, *Streptomyces*, *Microbacterium*, *Buchnera*, and *Candidatus* (Table S1). Many of these genera have been shown to be highly effective against diverse plant pathogens. As maize kernels were surface sterilized prior to the experiment, it can be assumed that the bacterial genera identified in this study are likely to be endophytes. A higher abundance (in absence of the pathogen) of specific endophytes such as *Streptomyces* and *Microbacterium* (at day 0) might also indicate the active production of antimicrobials in the kernels of resistant lines derived from these beneficial bacteria. Bioactive compounds such as 6-prenylindole, 3-acetonylidene-7-prenylindolin-2-one, kakadumycin A, and ehinodermycin isolated from *Streptomyces* have been experimentally validated for their antimicrobial properties against fungal (e.g., *Fusarium sp*.) and bacterial (e.g., *Staphylococcus sp*.) pathogens [20,23,24]. High endoglucanase activity in *Microbacterium* has been attributed to its antimicrobial properties besides other plant growth-promoting effects of this genus [25,26]. It will be interesting to see if any of these compounds can be detected in the kernels of resistant lines using sophisticated analytical methods such as liquid chromatography–mass spectrometry (LC–MS) in the future. Among the different treatment groups, infected kernels of the resistant line MI82 showed a significant increase in the population of *Sphingomonas* both at day 3 and day 7 or the relative abundance of *Stenotrophomonas* at day 3 compared to mock or infected kernels of the susceptible line SC212, respectively (Table S2). Both these bacterial endophytes have been reported to demonstrate antimicrobial properties, including antifungal activity in diverse plant species [27,28]. *Stenotrophomonas maltophilia* isolated from the medicinal plant *Fagonia indica* showed higher amounts of total flavonoids, antioxidant capacity, and antimicrobial activities against fungal and bacterial pathogens [29]. In rice (*Oryza sativa* L.), it was demonstrated that *Sphingomonas melonis* (*S. melonis*) accumulated in the disease-resistant rice seeds and was transmitted to next generations [30]. Further investigation showed that anthranilic acid produced by *S. melonis* interfered with the sigma factor (RpoS) of the seed-borne pathogen *Burkholderia plantarii*, leading to the impairment of virulence factor biosynthesis of the pathogen. The presence of these two endophytes may also highlight a temporal response to disease agents in the resistant lines, with *Stenotrophomonas* being an early responder and *Sphingomonas* being both an early and late responder during kernel infection. Interestingly, in the current study, representatives of the genera *Stenotrophomonas* and *Sphingomonas* were present in all genotypes, but their population increased significantly following fungal infection in the MI82 line only. This might indicate a role of the host plant’s genetic background in the regulation of levels of beneficial bacterial populations during pathogen infection [31] or recruitment of more efficacious strains of different or the same genera in resistant compared to susceptible lines. Significant shifts in the overall microbial community structure or composition in the resistant vs. susceptible maize lines were only observed in specific treatment groups (Figure 3 and Figure 4 and Figure S1). Principle coordinate analysis of samples originating from different treatments in the current study showed that infected kernels of the resistant line MI82 at day 7 were relatively clustered together than the rest (Figure 4A). The data presented here might also indicate the role of specific bacteria belonging to phyla such as *Proteobacteria*, *Alphaproteobacteria*, and *Enterobacteriales* in *A. flavus* resistance (Could these be potential biomarkers?), as evident in the infected kernels of MI82 at the early infection stage (day 3-I-M) (Figure S3). The kernels accumulated significantly lower aflatoxins (vs. SC212) at this time point [22]. Among the two different resistant lines though, MI82 had more specific trends in relation to relative abundance of putative beneficial bacteria while aflatoxin resistance in the resistant TZAR102 line may primarily be due to other host-associated factors [22].

Endophytes (bacteria and fungi) act as reservoirs of naturally occurring metabolites with diverse functions including their role in overall plant health and productivity [20]. In silico functional analysis of bacterial communities that showed significantly higher levels of relative abundance in resistant maize lines compared to the susceptible line suggests that antifungal activity may be associated with metabolites such as type II polyketides and sesquiterpenoids as well as the production of proteins demonstrating homology to neuroactive ligand–receptors and pancreatic secretion. Polyketides and sesquiterpenoids from beneficial microbes are well known for their antimicrobial properties [32,33]. The role of polyketides in mycotoxin control has been reported in maize [16]. A type II polyketide, fusaricidin, isolated from the maize kernel endophyte *Paenibacillus polymyxa* showed antifungal activities against *Fusarium graminearum* and reduced production of the mycotoxin deoxynivalenol (DON). Our data also show the presence of *Paenibacillus* in all the genotypes, though no significant differences were observed in *Paenibacillus* populations between the resistant and susceptible lines used in this study. This may suggest that the levels of resistance to *A. flavus* and aflatoxin contamination observed in the maize-resistant lines [22] were due to genotype-specific endophytic profiles that may include bacterial genera such as *Streptomyces* and *Microbacterium* (Table S2) capable of producing type II polyketides and sesquiterpenoids with antimicrobial properties [23,34]. In addition to polyketides derived from endophytic bacteria, polyketides produced by endophytic fungi have also demonstrated antimicrobial activities against fungal pathogens. Extracts from Amazonian plant endophytic fungal strains of *Talaromyces* identified antimicrobial compounds such as polyketides, steroids, anhydrides, and phenolics [35]. In vitro antimicrobial evaluation of these compounds demonstrated the antifungal properties of specific polyketides. Though not documented for their potential role in antimicrobial resistance or beneficial roles in plants, proteins were identified demonstrating homology to neuroactive ligand–receptors and in pancreatic secretion (Figure S4) that have been implicated in stress adaptation in mammals that consume corn as food [36]. Further genomic and metabolomic functional studies will be required for these two classes of genes as well as others identified in our in-silico analyses to better determine if they are in fact involved in host plant resistance to pathogens.

Future functional characterization of potential bacterial endophytes such as *Stenotrophomonas* and *Sphingomonas* for anti-*A. flavus* and anti-aflatoxigenic properties either through isolation from infected kernels of resistant lines or already available strains from the same genus will assist in the development of novel biocontrol agents targeting *A. flavus*. It will be useful to examine if specific microbiome-related genera associated with aflatoxin resistance in the resistant maize lines can be transferred through breeding to susceptible genotypes that do not carry these beneficial endophytes naturally. In addition, candidate biocontrol endophytes can also be examined for efficacy following seed or foliar application of susceptible genotypes in the field and if vertical transfer (seed to seed) of beneficial endophytes is possible in *A. flavus*-susceptible maize genotypes. Furthermore, the use of maize cultivars with seed endophytes perhaps may not only confer resistance to *A. flavus* infection but also improve protection against other abiotic and biotic stresses.

## 4. Materials and Methods

### 4.1. Maize Kernel Inoculation and Incubation

Maize kernels that were undamaged and were uniform in size were collected from the *A. flavus*-susceptible genotype SC212 and resistant genotypes TZAR102 (highly resistant) and MI82 (moderately resistant) [37] and were used in the kernel screening assay (KSA [38]) as reported earlier [18]. The resistant lines used in this study were characterized earlier for significantly lower surface growth of fungal mycelia and lower accumulation of aflatoxin than the susceptible line. Maize kernels were surface sterilized using 70% ethanol followed by air drying and stored under sterile conditions prior to the start of experiments. A highly pathogenic and high aflatoxin-producing *A. flavus* AF13 strain (isolate SRRC 1532 [39]) was grown on V8 agar medium for 7 days at 30 °C under illumination, and the spores were collected for kernel inoculation. Surface-sterilized kernels were placed in a sterile 300 mL beaker that contained 100 mL of spore suspension (4 × 10^6^ spores/mL) and continuously stirred for 3 min. After removing any excess inoculum, the kernels were placed in plastic caps that were arranged in trays containing moist filter paper on the bottom. The filter paper was kept moist by adding sterile ddH_2_O during the course of the experiment in order to maintain high relative humidity. Kernels inoculated with *A. flavus* or water (control, “mock-inoculated”) were placed inside trays (with lids on top). The trays were placed in an incubator in the dark at 31 °C. The kernels were collected at day 0, day 3, and day 7 post-inoculation. The samples were flash frozen using liquid N and stored at −80 °C prior to further processing using a Geno/Grinder (Metuchen, NJ, USA). Each treatment had three biological replicates, and each biological replicate consisted of four kernels.

### 4.2. Genomic DNA Extraction, PCR Amplification, and 16S rRNA Sequencing

Genomic DNA (gDNA) was extracted from ground maize kernel samples using the DNeasy PowerFood Microbial Kit (Qiagen, Valencia, CA, USA) following manufacturer’s protocol and stored at −20 °C. The extracted DNA were then used to set up PCRs. The 16S rRNA primer pair, 341F CCTACGGGNGGCWGCAG and 785R GACTACHVGGGTATCTAATCC [40], was utilized to evaluate the microbial ecology of each sample with methods via the bTEFAP^®^ DNA analysis service with peptide nucleic acid (PNA) blocking [41]. Amplicon sequencing using next generation technology (bTEFAP^®^) was originally described by Dowd et al. [42] and has been utilized in describing a wide range of environmental and gut microbiomes [43,44,45,46,47]. A reengineered version of bTEFAP^®^ has become one of the most widely published methods for evaluating microbiota and has been adapted to current next generation sequencing (NGS) technologies such as the Ion S5, Illumina’s MiSeq, HiSeq, and NovaSeq platforms as well as the PacBio Sequel.

Each sample underwent 35 cycle PCR using the HotStarTaq Plus Master Mix Kit (Qiagen, Valencia, CA, USA) under the following conditions: 95 °C for 5 min; followed by 35 cycles of 95 °C for 30 s, 75 °C for 10 s, 53 °C for 40 s, and 72 °C for 1 min; and after which a final elongation step at 72 °C for 10 min was performed. Following PCR, all amplicon products from different samples were mixed in equal concentrations and purified using calibrated Solid Phase Reversible Immobilization (SPRI) beads (Bulldog Bio, Inc., Portsmouth, NH, USA). The samples were sequenced utilizing the Illumina MiSeq (Illumina Inc., San Diego, CA, USA) chemistry following manufacturer’s protocols.

### 4.3. Data Processing

The metagenomic sequence reads were summarized and analyzed using the MR DNA ribosomal and functional gene analysis pipeline (MR DNA, Shallowater, TX, USA; www.mrdnalab.com, accessed on 12 March 2021). The MR DNA workflow utilized the Qiime2 microbiome bioinformatics pipeline [48] for the microbiome data analysis. This workflow is based on (i) quality assessment and filtering of reads through removing of short sequences <150 bp and ambiguous base calls using a maximum expected error threshold of 1.0; (ii) classifying unique sequences after removing sequencing or PCR point errors and chimera sequences; (iii) dereplication, whereby all identical sequences were combined into unique sequence reads; and (iv) assignment of the zero-radius operational taxonomic unit (zOTU). Final zOTUs were taxonomically classified using BLASTn against a curated database derived from National Center for Biotechnology Information (NCBI; www.ncbi.nlm.nih.gov, accessed on 12 March 2021) and compiled into each taxonomic level.

By using zOTU abundance tables, beta diversity was estimated using principal coordinate analysis, and Weighted UniFrac distances and the Shannon Diversity Index were used for alpha diversity [49,50]. Additionally, the dual hierarchical dendogram was used to display the abundance pattern of most representative bacterial communities among treatments [51]. Linear discriminant analysis effect size (LEfSe) was applied for identification of any differentially abundant families among treatments for potential biomarker discovery [52]. The relative abundance of taxonomic features classified using the greengenes 13_8 database was used as input for LEfSe analysis using the huttenhower server (https://huttenhower.sph.harvard.edu/galaxy/, accessed on 12 March 2021). The default LDA effect size alpha value (*p* = 0.05) and default LDA score (2.0) were used to identify significant differences between groups. Functional profiling of microbial communities was predicted using the Phylogenetic Investigation of Communities by Reconstruction of Unobserved States (PICRUSt) algorithm [53]. Closed reference OTUs were clustered using the greengenes 13_8 database. The resulting “.biom” file was used as input into PICRUSt (http://huttenhower.sph.harvard.edu/galaxy/, accessed on 12 March 2021). The input OTU table was then normalized, and final metagenome functional predictions were made using the Kyoto Encyclopedia of Genes and Genomes (KEGG) database [54,55] as a functional reference.

### 4.4. Statistical Analysis

Statistical analysis was performed using three statistical programs including XLSTAT (Addinsoft, New York, NY, USA), NCSS (NCSS, Kaysville, UT, USA), and “R” (https://www.R-project.org, accessed on 12 March 2021). Alpha and beta diversity analysis was conducted as described previously [42,43,56,57,58] using Qiime 2 [48]. Significance reported for any analysis is defined as *p* < 0.05.

## Data Availability

The authors declare that the data and material are available upon request.

## Supplementary Materials

The following are available online at https://www.mdpi.com/article/10.3390/ijms22073747/s1.
