# Supplementary material for: Changes in Bacterial Endophyte Community Following Aspergillus flavus Infection in Resistant and Susceptible Maize Kernels
Source: Int J Mol Sci. 2021 Apr 3;22(7):3747. doi: 10.3390/ijms22073747 (PMC8038446; doi:10.3390/ijms22073747)
Supplement: Supplementary file 1 [file ijms-22-03747-s001.pdf]

Table S1. Percentage of bacterial phyla detected in different treatment groups. Data are mean ± SE of 3 replicates, each replicate consists of four seeds. S: SC212; susceptible genotype, T: TZAR102; resistant genotype, M: MI82; resistant genotype.

Groups	tenericutes	proteobacteria	firmicutes	actinobacteria	bacteroidetes	chloroflexi	planctomycetes	aquificae	acidobacteria	euryarchaeota
Day0-S	85.96302876	13.83004548	0.089991816	0.061077311	0.054982859	0	0	0	0	0.000873778
Day0-T	98.60156332	0.201117003	0.129439758	0.980489472	0.074183169	0.001433897	0.010945534	0	0	0.000827847
Day0-M	99.75412981	0.165493058	0.05291371	0.025925015	0.000928744	0	0	0	0	0.000609664
Day3-Mock-S	99.71774862	0.219672075	0.01870194	0.030601328	0.009629192	0.00364685	0	0	0	0
Day3-Mock-T	96.86224327	2.564403012	0.51439926	0.052737029	0.005183625	0	0	0	0	0.001033803
Day3-Mock-M	98.6179636	1.340325534	0.018208217	0.020100084	0.003123923	0	0	0	0	0.000278646
Day7-Mock-S	99.15397859	0.182193253	0.325799515	0.333966069	0.003281126	0	0.000260484	0	0	0.000520968
Day7-Mock-T	99.83226407	0.131875201	0.0100213	0.023878182	0.000970306	0.00025569	0	0.00025569	0.00025569	0.000223867
Day7-Mock-M	99.84589013	0.083045963	0.025916792	0.028255674	0.012428997	0.00184613	0	0.001801447	0.00031971	0.000495155
Day3-I-S	96.52262352	3.3918953	0.020334631	0.044591937	0.01314511	0.00381402	0	0.002389807	0.000601042	0.000604631
Day3-I-T	97.82054777	2.139368385	0.015358968	0.022769835	0.001102923	0	0.000278446	0	0	0.000573674
Day3-I-M	99.72889608	0.112891361	0.109639262	0.02367586	0.023084315	0.000294954	0.000819422	0.000294954	0	0.000403791
Day7-I-S	99.73438743	0.149825542	0.01286331	0.004104594	0.000989274	0.056087747	0	0.021879755	0.019581988	0.000280359
Day7-I-T	99.75198097	0.203889359	0.02479637	0.005753045	0.010785902	0.000245848	0.000245848	0.000245848	0	0.002056809
Day7-I-M	93.18602466	0.676035175	5.148698007	0.111282797	0.545237568	0.205585937	0.126248761	0	0	0.000887091

Table S2. Bacterial genera that were significantly different among different treatment groups (** pairwise comparison with $p<0.05$). Data are mean ± SE of 3 replicates, each replicate consists of four seeds. S: SC212; susceptible genotype, T: TZAR102; resistant genotype, M: MI82; resistant genotype; I: infected.

Genera	Day 0 T vs. S	Day 0 M vs. S	Day 3-T I vs. mock	Day 3-M I vs. mock	Day 3-S I vs. mock	Day 3-I T vs. S	Day 3-I M vs. S	Day 7-T I vs. mock	Day 7-M I vs. mock	Day 7-S I vs. mock	Day 7-I T vs. S	Day 7-I M vs. S
Stenotrophomonas				**								**
Sphingomonas				**								
Streptomyces	**											
Microbacterium	**											
Buchnera						**	**					
Candidatus	**	**										

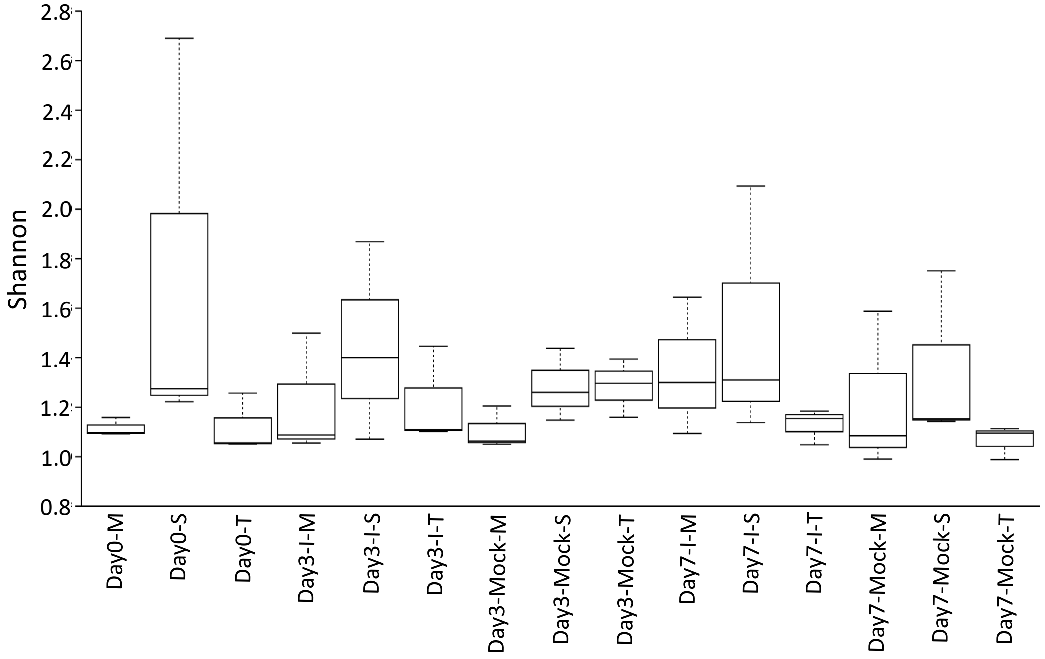

kruskal-wallis-pairwise-Groups		
Group 1	Group 2	p-value**
Day3-Mock-S	Day7-Mock-T	0.049534613
Day3-Mock-T	Day7-Mock-T	0.049534613
Day7-I-S	Day7-Mock-T	0.049534613
Day7-Mock-S	Day7-Mock-T	0.049534613

Figure S1. Shannon Diversity Boxplot and Kruskal-Wallis Pairwise Comparisons (**$p<0.05$). Data are mean ± SE of 3 replicates, each replicate consists of four seeds. S: SC212; susceptible genotype, T: TZAR102; resistant genotype, M: MI82; resistant genotype.

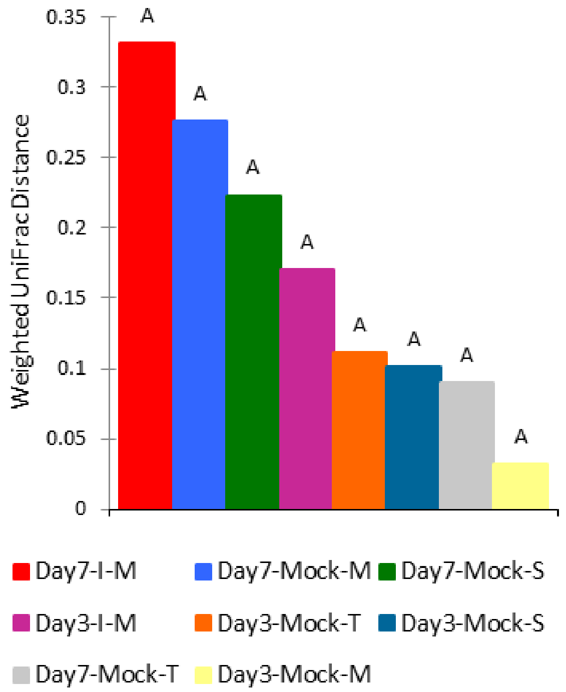

Figure S2. Comparison of within-group weighted unifrac distances. S: SC212; susceptible genotype, T: TZAR102; resistant genotype, M: MI82; resistant genotype. Mock = mock control, and I = *A. flavus* infected. Uppercase 'A' above the bars has been used to compare any significant difference among treatments. Data are mean ± SE of 3 replicates, each replicate consists of four seeds.

(A)

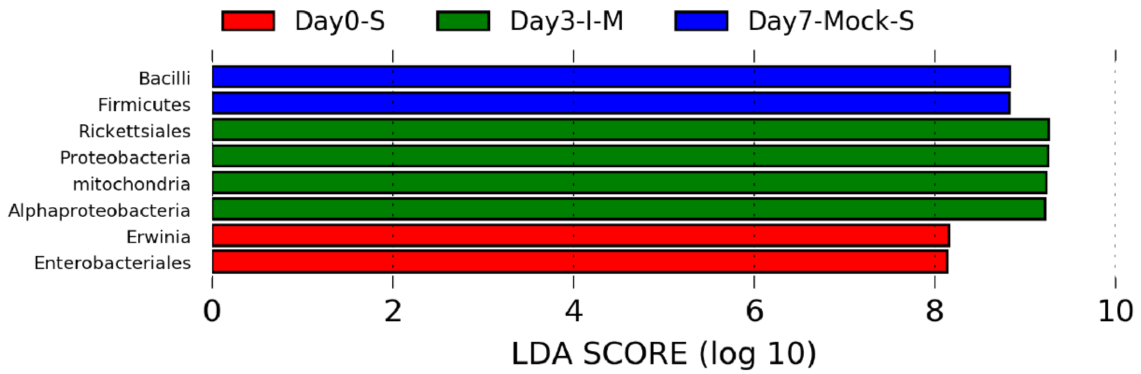

(B)

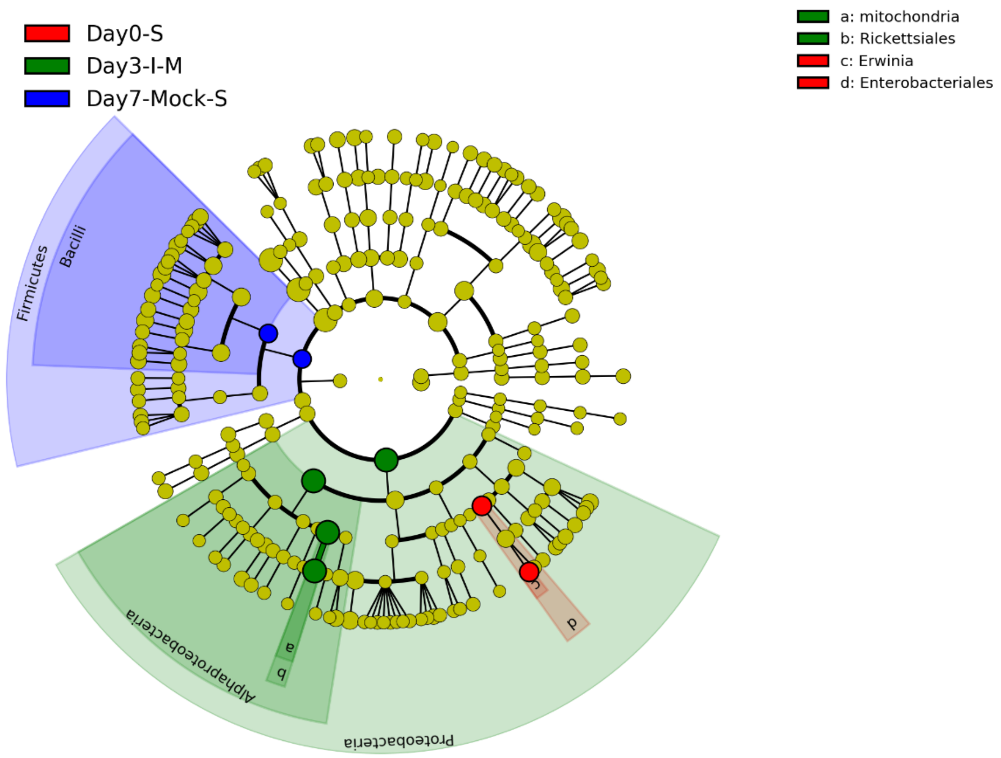

Figure S3. LEfSe analysis of maize genotypes. (A) Bar plot of statistically significant ($p < 0.05$) discriminative features among *A. flavus* susceptible and resistant maize genotypes; and (B) Hierarchal taxonomic cladogram displaying significant biomarkers in *A. flavus* susceptible and resistant maize genotypes. The diameter of each circle is proportional to the abundance of that specific taxon. The cladogram structure spans from phylum to genus, with phylum located nearest the center and increasing in taxonomic level with each progressive ring. S: SC212; susceptible genotype, T: TZAR102; resistant genotype, M: MI82; resistant genotype. Mock = mock control, and I = *A. flavus* infected. Data are mean ± SE of 3 replicates, each replicate consists of four seeds.

(A)

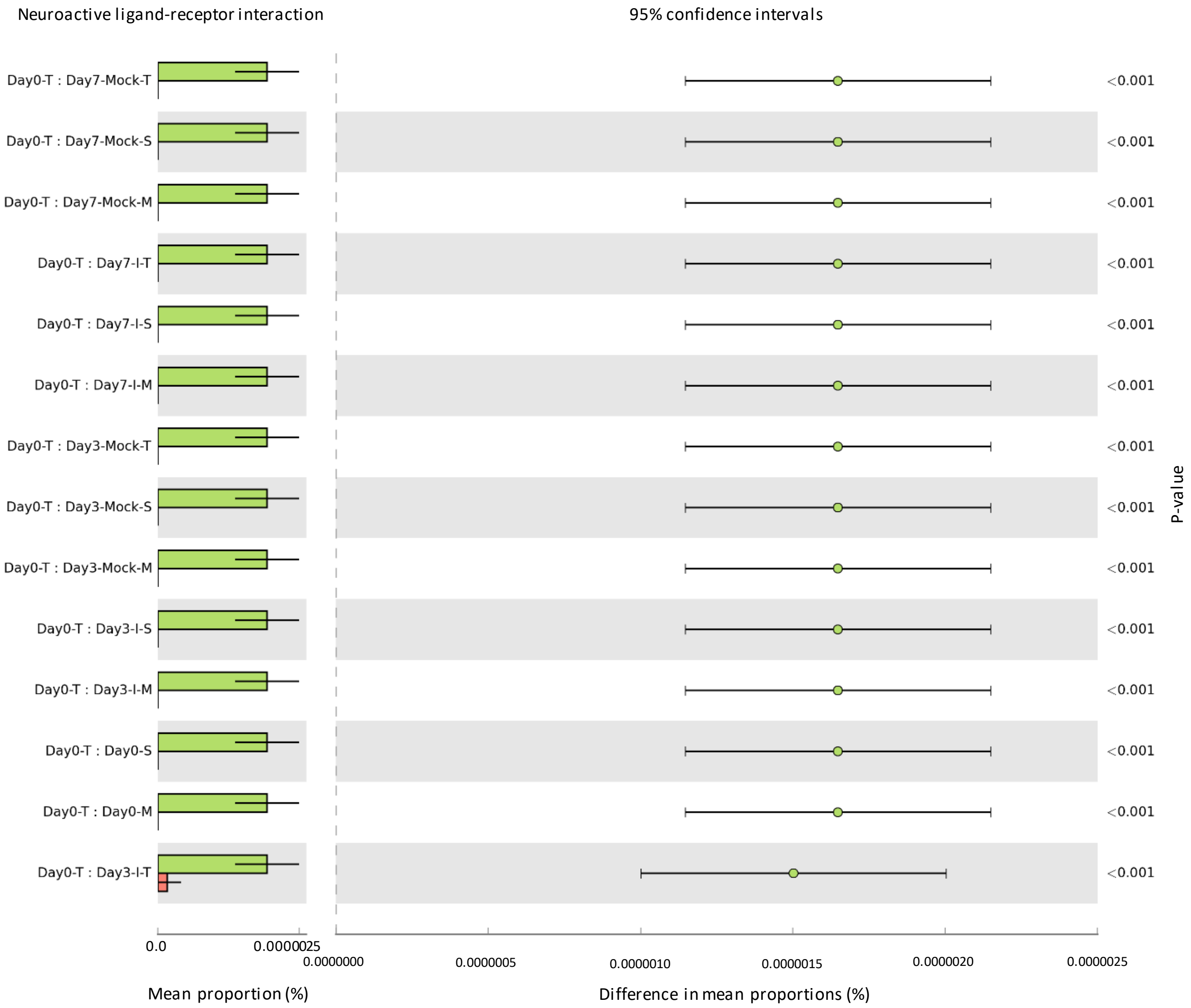

Figure S4. (continued)

(B)

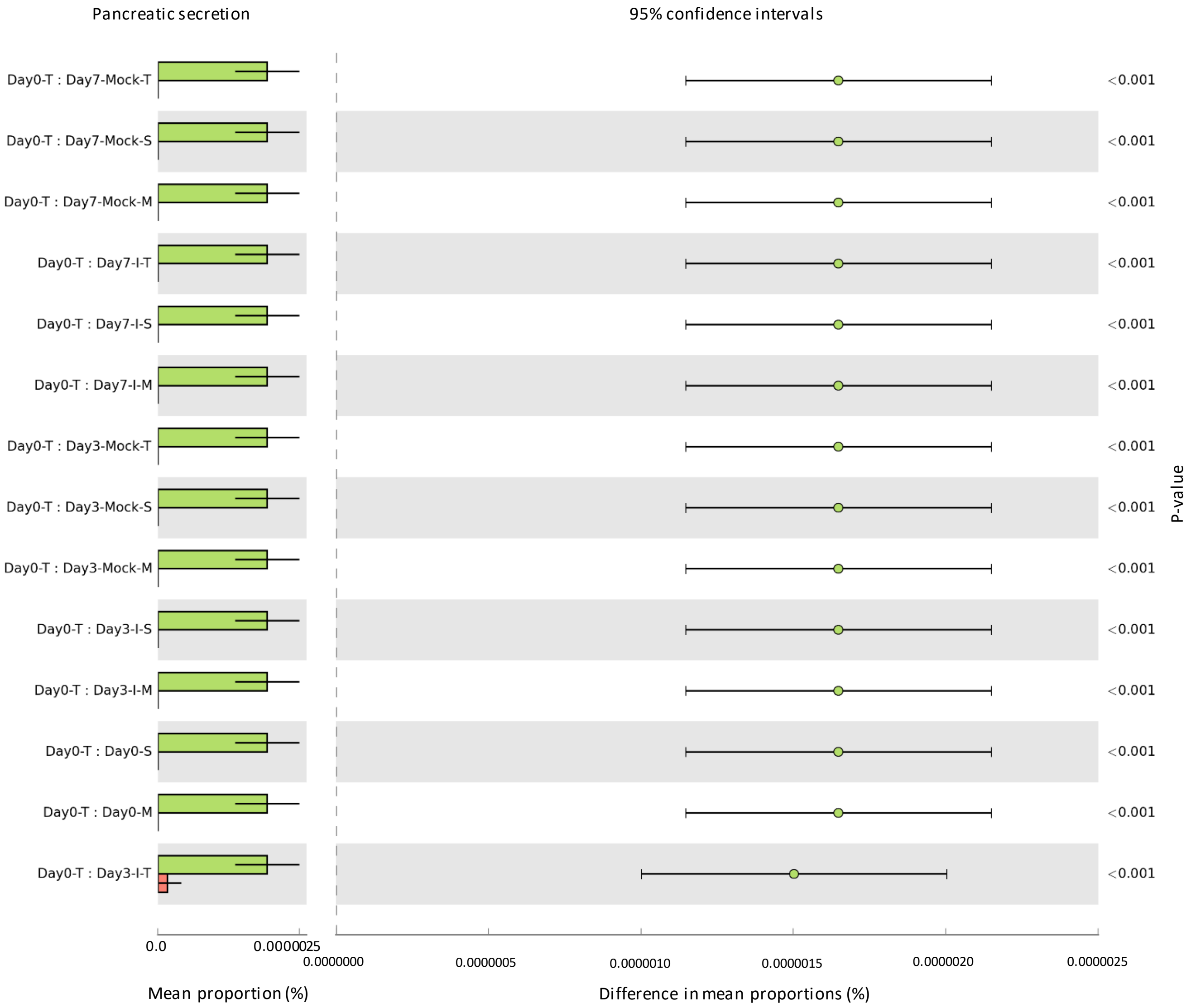

Figure S4. KEGG modules found to be significantly different ($p<0.05$) between treatment groups after Bonferroni test correction. (A) Neuroactive ligand-receptor interaction; (B) Pancreatic secretion and Sesquiterpenoid biosynthesis. Note: The expression of each KEGG module is significantly reduced between Day0-T (Green) and all other treatment groups. S: SC212; susceptible genotype, T: TZAR102; resistant genotype, M: MI82; resistant genotype. Mock = mock control, and I = *A. flavus* infected. Data are mean ± SE of 3 replicates, each replicate consists of four seeds.
